# The effect of age on vertex-based measures of the grey-white matter tissue contrast in autism spectrum disorder

**DOI:** 10.1186/s13229-018-0232-6

**Published:** 2018-10-01

**Authors:** Caroline Mann, Anke Bletsch, Derek Andrews, Eileen Daly, Clodagh Murphy, MRC AIMS Consortium, Declan Murphy, Christine Ecker

**Affiliations:** 1 Department of Child and Adolescent Psychiatry, Psychosomatics and Psychotherapy, University Hospital, Goethe University Frankfurt am Main, Deutschordenstrasse 50, 60528 Frankfurt am Main, Germany; 2 Department of Psychiatry and Behavioural Sciences, The Medical Investigation of Neurodevelopmental Disorders (MIND) Institute, UC Davis School of Medicine, University of California Davis, Sacramento, CA USA; 3 Department of Forensic and Neurodevelopmental Sciences, and the Sackler Institute for Translational Neurodevelopmental Sciences, Institute of Psychiatry, Psychology & Neuroscience (IoPPN), King’s College London, London, SE5 8AF UK

**Keywords:** Autism spectrum disorder, Neurodevelopment, Structural MRI, Neuroimaging, Brain anatomy

## Abstract

**Background:**

Histological evidence suggests that autism spectrum disorder (ASD) is accompanied by a reduced integrity of the grey-white matter boundary. This has also recently been confirmed by a structural neuroimaging study in vivo reporting significantly reduced grey-white matter tissue contrast (GWC) in adult individuals (18–42 years of age) with ASD relative to typically developing (TD) controls. However, it remains unknown whether the neuroanatomical differences in ASD at the grey-white matter boundary are stable across development or are age-dependent.

**Methods:**

Here, we examined differences in the neurodevelopmental trajectories of GWC in a cross-sectional sample of 77 male ASD individuals and 76 typically developing (TD) controls across childhood and early adulthood (from 7 to 25 years).

**Results:**

Using nested model comparisons, we first established that the developmental trajectory of GWC is complex in many regions across the cortex and includes linear and non-linear effects of age. Second, while ASD individuals have significantly reduced GWC overall, these differences are age-dependent and are most prominent during childhood (< 15 years).

**Conclusions:**

Taken together, our findings suggest that differences in GWC in ASD are unlikely to reflect atypical grey matter cytoarchitecture alone, but may also represent other aspects of the cortical architecture such as age-dependent variability in myelin integrity.

**Electronic supplementary material:**

The online version of this article (10.1186/s13229-018-0232-6) contains supplementary material, which is available to authorized users.

## Background

Autism spectrum disorder (ASD) is a complex neurodevelopmental condition characterized by deficits in social communication, social reciprocity, and repetitive/stereotypic behaviour [1]. There is strong evidence to suggest that these core symptoms are accompanied by differences in grey matter (GM) neuroanatomy and white matter (WM) connectivity [2], which typically manifest during early infancy [3, 4]. Despite the large number of existing neuroimaging studies, however, the neurobiological mechanisms that drive the atypical development of the brain in ASD remain poorly understood.

To date, most neuroimaging studies examining atypical brain development in ASD have focused on measures of brain volume [5–7] and its two constituent components cortical thickness [8] and surface area [9, 10]. More recently, however, the attention of structural neuroimaging studies is shifting towards examining the grey-white matter boundary, as histological evidence suggests that the grey-white matter tissue contrast may be regionally less well defined (i.e. less distinct) in ASD [11]. Such ‘blurring’ of the grey-white matter transition zone seems to be caused by the presence of supernumerary neurons beneath the cortical plate, which—in turn—may result from migration deficits or failed apoptosis in the subplate region [12]. This finding also agrees with genetic investigations linking the aetiology of ASD to atypical neuronal proliferation, migration, and maturation [13, 14]. For stratification purposes, and to capture aspects of ASD neuropathology that may be more closely linked to aetiological factors, it is therefore important to also investigate neuroimaging measures that map onto these particular characteristics of the cortical microstructure in vivo.

With this aim in mind, we recently examined the contrast between grey and white matter (GWC) across different cortical layers in a sample of males and females with ASD and typically developing (TD) controls [15]. We found that the GWC was significantly reduced in ASD, particularly at the grey-white matter boundary, and in many brain regions that have previously been linked to autistic symptoms and traits [16]. Our in vivo finding of a reduced GWC is also consistent with prior *postmortem* reports of a less well-defined grey-white matter boundary in ASD [11, 12]. However, based on tissue contrast alone, it is not possible to disentangle whether the observed between-group effects are driven by (1) differences in grey matter cytoarchitecture, as suggested by the above histological studies, or by (2) local variations in myelin content. For instance, a recent neuroimaging study of typical ageing, examining a sample of healthy adults (with an age range of 20–84 years), suggests that the GWC typically declines with increasing age and most likely reflects local (i.e. region-dependent) age-related changes of myelin integrity in the superficial WM [17]. Thus, by studying the GWC in ASD across different developmental stages, it may be possible to gain in vivo insights into neurobiological processes that (1) should be completed around birth (e.g. migration deficits), (2) end during early childhood (e.g. apoptosis), and (3) that are ongoing (e.g. myelination). Here, we examined age-related changes in GWC in ASD individuals compared to TD controls during childhood and adolescence. In addition to between-group differences in GWC, the present study investigated age-by-group interactions in a cross-sectional sample of male individuals with ASD and matched TD controls using a spatially unbiased ‘vertex-wise’ approach (i.e. not restricted to regions of interest). We expected the differences in the contrast to be age-dependent (i.e. there are significant age × group interactions), which would suggest that differences observed during postnatal brain development are not exclusively driven by atypical grey matter cytoarchitecture.

Furthermore, it has previously been shown that the trajectory of brain maturation for different morphological features is complex and cannot adequately be captured by linear effects alone. For example, the trajectory of total brain volume seems to be U-shaped with an increase in volume during early childhood, a peak during adolescence, and a subsequent decline in volume [18]. There are also studies to suggest that there is considerable regional variation in the complexity of the normal developmental trajectory of cortical thickness, for example, which includes cubic, quadratic, and linear effects [19]. When examining age effects, it is therefore important to establish linear as well as non-linear effects, in order to adequately model the neurodevelopmental trajectory. While the typical neurodevelopmental trajectories are well established for measures of brain volume or cortical thickness, there is currently no comparable data for vertex-based measures of GWC. In the present study, we therefore examined linear, quadratic, and cubic effects of age in order to model the complex trajectory of the GWC in children and young adults between 7 and 25 years of age.

## Methods

### Participants

Eighty-two (82) right-handed males with ASD and eighty-two (82) TD controls, aged 7 to 25 years were recruited and assessed at the Institute of Psychiatry, Psychology, and Neuroscience, King’s College, London. Both groups were matched for age, handedness (all right-handed), and full-scale IQ. Exclusion criteria for all participants included (1) history of major psychiatric disorder, (2) head injury, (3) genetic disorder associated with autism (e.g. fragile X syndrome and tuberous sclerosis), or (4) any other medical condition affecting the brain function (e.g. epilepsy). Furthermore, individuals with a history of substance abuse (including alcohol) and individuals taking antipsychotic medication, mood stabilizers, or benzodiazepines were excluded from the study. A diagnosis of ASD was based on the International Statistical Classification of Diseases, 10th Revision (ICD-10) [20] research criteria, and subsequently confirmed using the Autism Diagnostic Interview-Revised (ADI-R) [21] to ensure that all participants with ASD met the criteria for childhood autism. All individuals with ASD had to reach ADI-R algorithm cutoffs in the three domains of impaired reciprocal social interaction, communication, and repetitive behaviours and stereotyped patterns, although failure to reach cutoff in one of the domains by one point was permitted (see Table 1). The Autism Diagnostic Observation Schedule (ADOS) [22] was used to assess current symptoms, but was not used as inclusion criterion. Overall intellectual ability was assessed using the Wechsler Abbreviated Scale of Intelligence (WASI) [23]. All participants had a full-scale IQ (FSIQ) greater than 70 and gave informed written consent in accordance with the ethics approval by the National Research Ethics Committee, Suffolk, England. The participants over 18 years of age (*n* = 37 individuals with ASD and *n* = 22 TD controls) were also part of a recent study by our group examining the GWC during adulthood [15].

**Table 1:** Participant demographics

	ASD (*n* = 77)	TD controls (*n* = 76)
Age (years)	17 ± 4 (7–25)	16 ± 4 (8–25)
FSIQ	107 ± 14 (70–140)	111 ± 10 (84–134)
ADI-R social	20 ± 5 (9–28)	–
ADI-R communication	15 ± 5 (7–24)	–
ADI-R repetitive behaviour	6 ± 3 (2–20)	–
ADOS total	9 ± 3 (3–19)	–
Total grey matter volume [cm^3^]	716.99 ± 58.12	726.57 ± 66.99
Total white matter volume [cm^3^]	473.66 ± 55.87	478.71 ± 54.49
Total brain volume [cm^3^]	1190.65 ± 113.99	1205.28 ± 121.48

*Note. FSIQ*, full-scale IQ; *ADI-R*, Autism Diagnostic Interview-Revised; *ADOS*, Autism Diagnostic Observation Schedule. Data expressed as mean ± standard deviation (range). There were no significant between-group differences in age, FSIQ, or global brain measures at *p* < 0.05 (two-tailed)

### Structural MRI data acquisition

For all 164 participants, high-resolution structural T1-weighted volumetric images were acquired at the Centre of Neuroimaging Sciences, Institute of Psychiatry, Psychology, and Neuroscience, London, UK. Images were obtained using a 3-Tesla GE Signa System (General-Electric, Milwaukee, WI) with full-head coverage, 196 contiguous slices (1.1-millimetre (mm) thickness, with 1.09 × 1.09 mm in-plane resolution), a 256 × 256 × 196 matrix, and a repetition time/echo time (TR/TE) of 7/2.8 milliseconds (ms) (flip angle = 20°, FOV = 28 cm). A (birdcage) head coil was used for radiofrequency transmission and reception.

### Cortical reconstruction using FreeSurfer

Each T1-weighted scan was initially screened for clinical abnormalities or large-scale motion artifacts by a radiologist. Two percent of scans (*n* = 3 participants) had to be excluded from the analysis due to insufficient quality. Models of the inner (i.e. white matter) surface and outer (i.e. pial or grey matter) surface were derived using FreeSurfer v5.3.0 (http://surfer.nmr.mgh.harvard.edu/). These well-validated and fully automated procedures have been extensively described elsewhere [24–26]. In brief, a single filled white matter volume was generated for each hemisphere after intensity normalization, skull stripping, and image segmentation using a connected components algorithm. Then, a triangular surface tessellation was generated for each white matter volume by fitting a deformable template. This resulted in a triangular cortical mesh for grey and white matter surfaces consisting of approximately 150.000 vertices (i.e. points) per hemisphere. The resulting surface models were visually inspected for reconstruction errors. Surface reconstructions with visible inaccuracies were further excluded and are not described in this study. Dropout rates due to surface reconstruction errors represented approximately 5% of the total sample (*n* = 8 participants) and were approximately equal between groups. This procedure resulted in a final sample size of 153 participants (*n* = 77 individuals with ASD and *n* = 76 TD controls).

### Grey-to-white matter tissue contrast and absolute tissue intensities

**Fig. 1:**
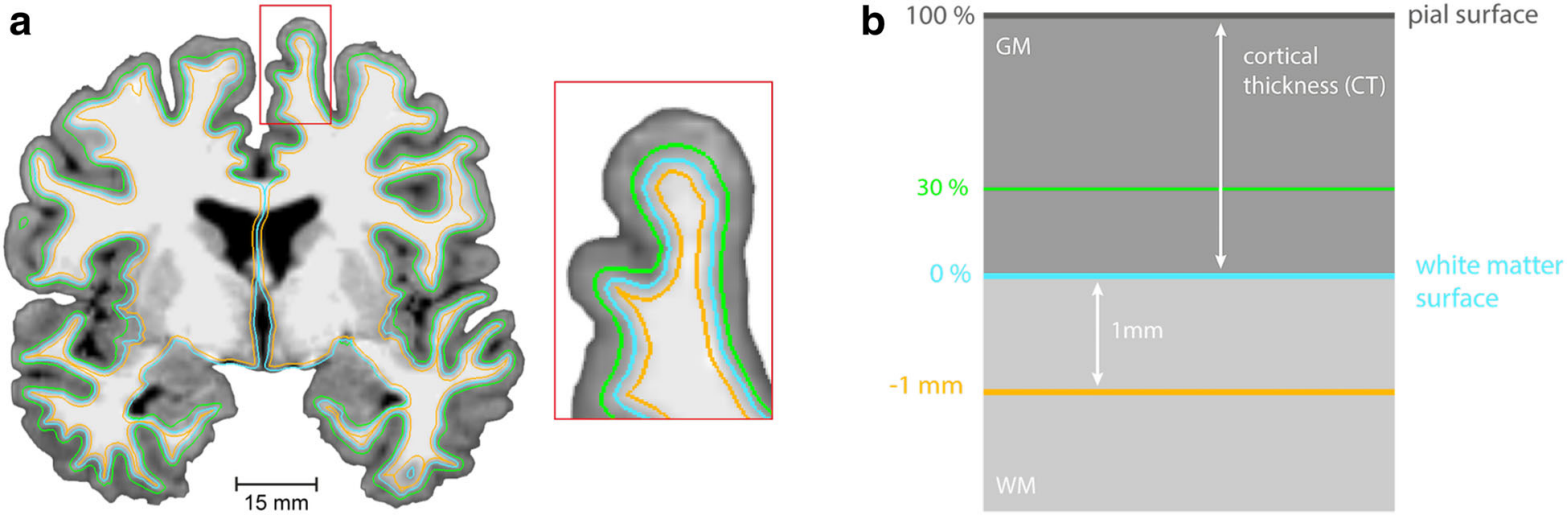
**a** Grey and white matter signal intensity sampling procedure. **b** At each cerebral vertex, GWC was calculated as the percentage of grey matter intensity (GMI) sampled at 30% cortical thickness (CT) relative to the white matter intensity (WMI) at 1 mm below the grey-white matter boundary. *Note. GM* grey matter, *WM* white matter

At each cerebral vertex (*i*), the grey-to-white matter tissue contrast (GWC) was calculated as the percentage of grey matter intensity (GMI) sampled at 30% cortical thickness (CT) relative to the white matter intensity (WMI) at 1 mm below the grey-white matter boundary (see Fig. 1), i.e.

$$ {\mathrm{GWC}}_i=\frac{100\times \left({\mathrm{WMI}}_{i,1\mathrm{mm}}-{\mathrm{GMI}}_{i,0.3}\right)}{0.5\times \left({\mathrm{WMI}}_{i,1\mathrm{mm}}+{GMI}_{i,0.3}\right)} $$

In a next analysis step, to determine the influence of grey and white matter intensity on the GWC, we also extracted the absolute grey (GMI) and white matter intensities (WMI) at each cerebral vertex following non-uniform (NU) intensity correction and normalization (i.e. scaling of mean intensity of the white matter to 110) of the images in FreeSurfer. Grey matter tissue intensities were sampled at a projection fraction of 0% CT (i.e. at the grey-white matter boundary), as well as at 30% CT. White matter tissue intensities were sampled at 1 mm into the white matter from the grey-white matter boundary (the FreeSurfer ‘default’ for the computation of the GWC). To improve the ability to detect population changes, the resulting GWC, GMI, and WMI overlays were smoothed using a 10-mm full-width at half-maximum (FWHM) surface-based Gaussian kernel prior to statistical analyses.

### Statistical analysis

Statistical analysis was conducted using the SurfStat toolbox (http://www.math.mcgill.ca/keith/surfstat/) for Matlab (R2016a; www.mathworks.com). To determine developmental trajectories for the GWC, we initially tested for linear, quadratic, and cubic effects of age, in addition to the main effect of group in a vertex-wise fashion. Here, an F-test for nested model comparisons was performed at each vertex, employing a step-up model selection procedure. Initially, the linear (i.e. most reduced) model was compared to a more complex (i.e. quadratic) model in order to determine if the addition of a quadratic age effect significantly improved the goodness-of-fit. If the quadratic model performed significantly better, it was then compared to the most complex (i.e. cubic) model, which contained a linear, quadratic, and cubic age term. Corrections for multiple comparisons across the whole brain were performed using random-field theory (RFT)-based cluster-corrected analysis for non-isotropic images using a *p* < 0.05 (two-tailed) cluster-significance threshold [27]. This procedure allowed us to identify the most parsimonious model at each vertex, i.e. the most simple plausible model that explained age-related variability in measures of GWC with the smallest set of predictors. All nested model comparisons were performed based on the combined sample of ASD and TD individuals.

Next, we examined between-group differences and age-by-group interactions by applying a general linear regression model (GLM) at each vertex *i* for subject *j*, with (1) group (G) as categorical fixed-effects factor and (2) linear, quadratic, or cubic terms for age, as well as their interactions with group. Based on previous reports suggesting that there is a significant negative association between the GWC and general cognitive abilities [28–31], FSIQ was included as continuous covariate, so that GWC_*i*_ *= β*_*0*_ *+ β*_*1*_*G*_*j*_ *+ β*_*2*_ age_*j*_ *+ β*_*3*_ age_*j*_^*2*^ *+ β*_*4*_ age_*j*_^*3*^ *+ β*_*5*_ (age_*j*_ × group_*j*_) *+ β*_*6*_ (age_*j*_^*2*^ × group_*j*_) *+ β*_*7*_ (age_*j*_^*3*^ × group_*j*_) *+ β*_*8*_ IQ_*j*_ *+ ε*_*i*_, where ε denotes the residual error. Corrections for multiple comparisons across the whole brain were performed as outlined above (i.e. using random-field theory (RFT)-based cluster-corrected analysis for non-isotropic images). All statistical effects (i.e. between-group differences and age-by-group interactions) were mapped onto the FreeSurfer high-resolution common-group template in standard space (i.e. ‘fsaverage’ with ~ 300.000 vertices). For reasons of completeness, we also performed the analysis using total brain volume (TBV) as a continuous covariate in the statistical model. The results of this analysis are presented in an additional file (see Additional file 1).

## Results

### Demographics

There were no significant between-group differences in age [*t* (151) = 1.32, *p* = 0.19], FSIQ [*t* (151) = − 1.76, *p* = 0.08], total grey matter volume [*t* (151) = − 0.94, *p* = 0.35], or total white matter volume [*t* (151) = − 0.57, *p* = 0.57] (see Table 1). We therefore did not covary for total brain measures in the statistical analysis of GWC, GMI, or WMI.

### Nested model comparison

**Fig. 2:**
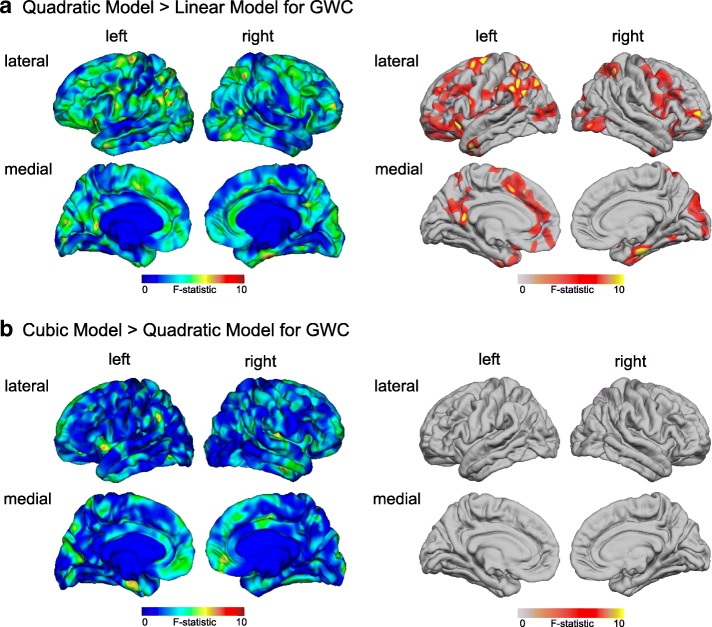
Nested model comparisons of GWC age effects mapped onto FreeSurfer default common group template (‘fsaverage’). **a** Comparison of the linear vs. quadratic model including all age effects and age-by-group interactions. **b** Comparison of the quadratic vs. cubic model including all age effects and age-by-group interactions. *Left panel* shows the difference map resulting from the model comparison. *F-*values (blue to red) indicate voxels where the more complex model provided a better fit than the more reduced model (*F*-statistic, unthresholded). *Right panel* indicates random-field theory (RFT)-based, cluster-corrected (*p* < 0.05) difference maps in goodness of fit (based on *F-*statistic). Here, the colourscale relates to *F*-statistic within significant clusters where the quadratic model provided a significantly better fit than the linear model

Based on the nested model comparison, we established that the quadratic model provided a significantly better fit than the linear model in several clusters across the cortex when modelling neurodevelopmental trajectories in GWC (see Fig. 2a). There were no brain regions which showed a significant improvement in fit when also including a cubic term for age (RFT-based, cluster-corrected, *p* < 0.05) (see Fig. 2b). Thus, the quadratic model was chosen as the most parsimonious model for the examination of age-related differences in GWC between ASD individuals and TD controls.

### Between-group differences in grey-to-white matter tissue contrast

**Fig. 3:**
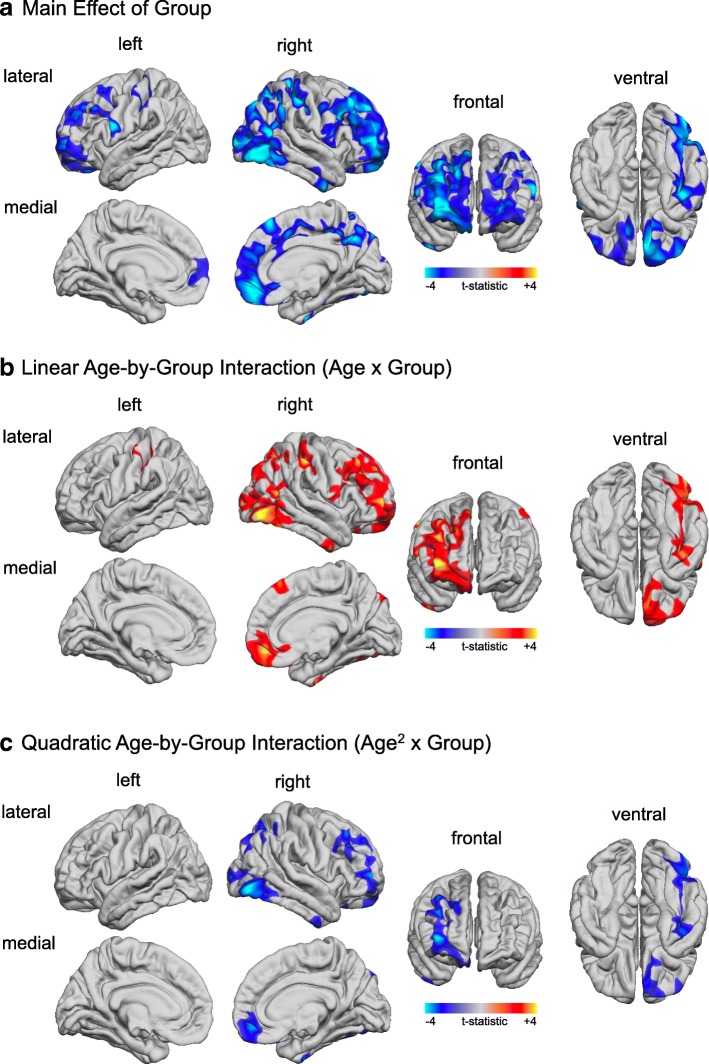
Between-group differences and age-by-group interactions for GWC. **a** Clusters with significantly reduced GWC (RFT-based, cluster corrected, *p* < 0.05) in ASD compared to controls (blue to cyan colourscale) while controlling for the effects of age and age-related interactions (i.e. main effect of group). **b** Clusters with significant linear age-by-group interactions (RFT-based, cluster corrected, *p* < 0.05). **c** Clusters with significant quadratic age-by-group interactions (RFT-based, cluster corrected, *p* < 0.05). *Note*. Significant positive age-by-group interactions are displayed in red to yellow, significant negative age-by-group interactions are displayed in blue to cyan

Overall, we found that the GWC was significantly reduced in ASD individuals relative to controls in seven large clusters following correction for multiple comparisons (RFT-based, cluster-corrected, *p* < 0.05, two-tailed). These clusters predominantly included (1) bilateral prefrontal cortices (approximate Brodmann area [BA] 6/10), (2) the right inferior parietal cortex (BA 39), (3) the right postcentral gyrus (BA 1/2), (4) the right precuneus (BA 7), and (5) the left supramarginal gyrus (BA 40, see Fig. 3a; for detailed statistical values see Table 2). There were no brain regions where ASD individuals showed a significant increase in GWC compared to TD controls.

**Table 2:** Clusters of significant reductions in GWC and WMI in ASD relative to controls

	Cluster	Region labels	Side	BA	Vertices	*t*_max_	*p*	Age (age^2^)-by-group interactions
GWC	1	Rostral middle frontal gyrus, superior frontal gyrus, precentral gyrus	R	10	24,950	− 3.96	3.17 × 10^−6^	Age (age^2^)
	2	Inferior parietal cortex, lateral occipital cortex, superior parietal cortex	R	39	20,502	− 4.10	3.17 × 10^−6^	Age (age^2^)
	3	Postcentral gyrus, superior parietal cortex, supramarginal gyrus	R	1/2	6528	− 4.22	3.49 × 10^−5^	Age (age^2^)
	4	Supramarginal gyrus, postcentral gyrus, inferior parietal cortex	L	40	4828	− 3.84	.00026	Age
	5	Caudal middle frontal gyrus, superior frontal gyrus, rostral middle frontal gyrus	L	6	5778	− 3.78	.00046	–
	6	Rostral middle frontal gyrus, superior frontal gyrus, lateral orbital frontal cortex	L	10	5243	− 3.18	.0036	–
	7	Precuneus cortex, paracentral lobule	R	7	3448	− 3.14	.037	–
WMI	1	Inferior parietal cortex, precuneus cortex, superior parietal cortex	R	39	7320	− 3.77	2.27 × 10^−6^	Age (age^2^)
	2	Rostral middle frontal gyrus, caudal middle frontal gyrus, pars orbitalis	R	9	5219	− 3.48	2.95 × 10^−5^	Age (age^2^)
	3	Supramarginal gyrus, inferior parietal cortex, superior temporal gyrus	L	40	7096	− 3.75	.0003	Age (age^2^)
	4	Lateral occipital cortex, inferior temporal gyrus, lingual gyrus	L	37	5296	− 3.91	.0005	Age (age^2^)
	5	Precuneus cortex, isthmus-cingulate cortex	L	7	1656	− 3.82	.0018	Age
	6	Lateral orbital frontal cortex	R	47	1052	− 4.25	.022	–
	7	Lateral occipital cortex, inferior temporal cortex, middle temporal gyrus	R	19	2907	− 3.07	.04	–
	8	Fusiform gyrus	R	37	1568	− 3.27	.048	–

*Note. GWC*, Grey-white matter contrast; *WMI*, white matter intensity; *BA*, approximate Brodmann area at *t*_max_ within cluster; *L*, left; *R*, right; *t*_max_, test statistic within cluster; *p*, cluster-corrected *p* value. Vertices: the number of vertices within the cluster; age/age^2^ indicates existence of significant age-by-group interaction for linear and/or quadratic terms. For each cluster, the three most significant regions are reported

### Significant age-by-group interactions in grey-to-white matter tissue contrast

**Fig. 4:**
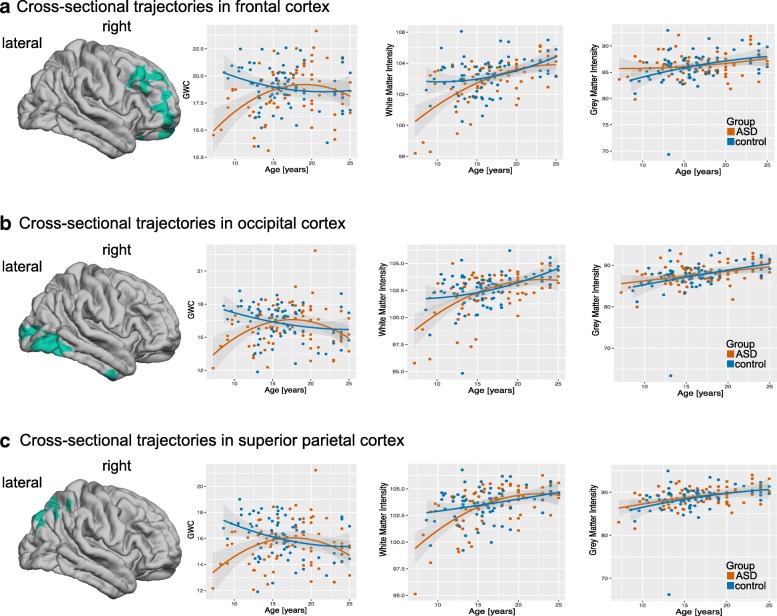
Developmental trajectories for GWC, WMI, and GMI in clusters with observed significant quadratic age-by-group interactions (see Fig. 3c). **a** Quadratic age-by-group interaction in the cluster located on the right dorsolateral prefrontal cortex (DLPFC) (cluster 1 in Table 2). **b** Quadratic age-by-group interaction in the cluster located on the right lateral occipital cortex (cluster 2 in Table 2). **c** Quadratic age-by-group interaction in the cluster located on the right superior parietal cortex (cluster 3 in Table 2)

In four of the seven clusters with a significant between-group difference in GWC, we also observed significant age-by-group interactions in addition to the main effect of group. Linear age-by-group interactions were observed in (1) the right inferior parietal cortex (BA 39), (2) the right prefrontal cortex (BA 10), (3) the right postcentral gyrus (BA 1/2; see Fig. 3b), and (4) the left supramarginal gyrus (BA 40). In addition, three clusters in the right hemisphere that included frontal, parietal, and temporal regions displayed a significant quadratic interaction (i.e. age^2^-by-group) (see Fig. 3c). In brain regions with significant age-by-group interactions, individuals with ASD tended to have the most prominent decrease in GWC during childhood and early adolescence (i.e. between 7 and 15 years of age) as compared to TD controls, but showed no differences (or enhanced GWC) during early adulthood (see Fig. 4). There were no clusters with significant linear or quadratic age-by-group interactions that did not also have a significant main effect of group, i.e. all clusters with significant age-by-group interactions also displayed a significant main effect of group.

### Between-group differences in grey matter intensities and white matter intensities

**Fig. 5:**
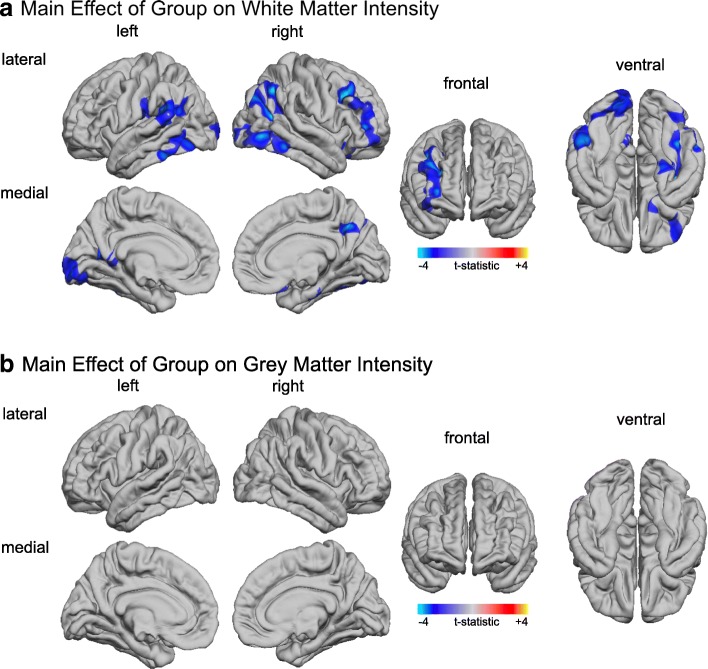
Between-group differences for absolute grey and white matter tissue intensities (GMI, WMI respectively). **a** Clusters with significantly reduced WMI (blue to cyan colourscale) at 1 mm below the white matter surface (RFT-based, cluster-corrected, *p* < 0.05) in ASD compared to controls while controlling for the effects of age and age-related interactions (i.e. main effect of group). **b** Between-group differences for GMI at 30% cortical thickness (RFT-based, cluster corrected, *p* < 0.05). The colourbar shows *t-*statistics resulting from the main effect of group. Significant positive age-by-group interactions are displayed in red to yellow, and significant negative age-by-group interactions are displayed in blue to cyan

In addition to the GWC, we also examined between-group differences in absolute grey and white matter tissue intensities (i.e. GMI and WMI) in order to identify whether the between-group differences in GWC were driven by variability within the cortical grey or white matter or a combination of both. We did not observe any significant between-group differences in absolute tissue intensities when sampling at 0% (i.e. at the grey-white matter boundary) or at 30% into the grey matter (*p* > 0.05, two-tailed). However, individuals with ASD had significantly decreased WMI relative to controls in many brain regions that also showed decreases in GWC. These regions included (1) bilateral lateral occipital cortices (approximate Brodmann area [BA] 37/19), (2) the right prefrontal cortex (BA 9/47), (3) the right inferior parietal cortex (BA 39), (4) the right fusiform gyrus (BA 37), (5) the left supramarginal gyrus (BA 40), and (6) the left precuneus (BA 7; see Fig. 5; for detailed statistical values, see Table 2). There were no brain regions where ASD individuals showed a significant increase in WMI compared to TD controls. Thus, our data suggests that in children and adolescents with ASD, between-group differences in WMI underneath the cortical mantle contribute more to differences in the GWC than do differences in GMI.

## Discussion

In the present study, we examined between-group differences in cross-sectional age-related trajectories of GWC in ASD and neurotypical controls across childhood and early adulthood (from 7 to 25 years) using a spatially unbiased vertex-wise approach. We first established that the developmental trajectory of GWC is complex in many areas of the brain and included linear as well as non-linear (i.e. quadratic) effects of age. Moreover, we found that while ASD individuals had significantly reduced GWC overall, these differences were age-dependent, with the most prominent decreases in GWC occurring during childhood. This is of importance as our findings suggest that differences in GWC in ASD are unlikely to reflect atypical grey matter cytoarchitecture alone, which is typically set around birth, but may also represent age-related variability in the white matter architecture (i.e. differences in myelination, axonal density, and diameter, etc.). Measures of GWC might thus be considered an age-sensitive in vivo marker for atypical neurodevelopment in ASD. Our finding of significantly reduced GWC in children and adolescents with ASD extends our previous neuroimaging study examining the GWC in adults with the condition, which concluded in suggesting that the tissue contrast between cortical grey and white matter may be less well defined in ASD [15].

Moreover, our study agrees with previous post-mortem reports suggesting that the boundary between cortical layer VI and the underlying white matter may be more ‘indistinct’ in ASD. This indistinct boundary may be due to increased ‘dispersion’ of neuronal cells across the grey-white matter interface [11, 12]. In turn, supernumerary neurons beneath the cortical plate may then arise as a consequence of disrupted migratory processes during prenatal brain development and/or atypical development and resolution of the cortical subplate (e.g. overproduction of subplate neurons or reduced apoptosis) [32]. Additionally, the cortical suplate plays a crucial role in the formation of the early intra- and extra-cortical neurocircuitry and contributes to the guidance and targeting of thalamocortical axons [33].

Significant reductions in GWC were found in several regions across the cortex, most of which have previously been associated with symptoms characteristic for ASD. More specifically, the medial and dorsolateral prefrontal cortices (mPFC and DLPFC) are integral parts of the so-called social and emotional brain, which encompasses a set of brain regions involved in wider aspects of social cognition and emotional processing [34, 35]. ASD-related neuroanatomical variation in these regions has also been linked to deficits in theory of mind [36], face processing [37], and various other aspects of impaired social cognition, for example, self-referential cognition and empathy [38]. In addition, some of our identified clusters (mPFC and precuneus) are also an integral component of the so-called default mode network (DMN), which characterizes a wider network of brain regions showing decreased activity during cognitive tasks and increased activity when the brain is ‘at rest’ [39]. In ASD, the DMN has been reported to be among the most disrupted functional networks, and this disrupted intrinsic DMN organization (e.g. in terms of functional connectivity patterns) seems to be associated with social deficits in children and adults with ASD [40]. Further significant reductions in GWC were found in occipital regions, which on a functional level have been associated with communication deficits and social reciprocity [2].

In many brain regions where we found a significant main effect of group in GWC, we also observed significant linear and quadratic age-by-group interactions. This implies that the between-group differences in these regions are age-dependent and caused by an atypical developmental trajectory of GWC in the ASD individuals. More specifically, while the GWC in TD controls declined consistently from 7 to 25 years of age, the cross-sectional age-related trajectory in ASD was significantly decreased relative to the normative trajectory during early childhood, followed by a period of no, or small, differences between the ages of 15 and 23 years. This early age-related reduction in tissue contrast was most prominent in temporal and prefrontal regions, which are also the latest ones to mature during typical development [41]. Given previous evidence to suggest that the GWC declines significantly as part of the typical ageing process [17, 42], and based on our previous results of a reduced GWC in adults with ASD [15], it is likely that the GWC also declines more rapidly across the remaining life-span (i.e. after the age of 23 years). However, future research is needed to test this hypothesis directly, using samples with a wider age range (i.e. 25 years plus). Taken together, our study suggests that the developmental trajectory of GWC in the ASD brain not only differs quantitatively from the trajectory in TD controls, but also qualitatively (i.e. in terms of its shape), and particularly during childhood. In turn, this implies that the GWC in ASD may be mediated via different neurobiological mechanisms as compared to TD controls.

Notably, our results show a lateralization towards the right hemisphere, i.e. group differences in GWC were mostly located in the right hemisphere while the left hemisphere seems to be relatively unimpaired. Previous studies have yielded highly heterogeneous findings concerning the lateralization of structural and functional abnormalities in the brain in ASD (e.g. [43]). On the functional level, studies demonstrate that the right hemisphere in particular seems to play a crucial role in mediating several autistic core symptoms, such as communication [44] and theory of mind deficits [45]. Furthermore, in a study by Dapretto et al. [46], ASD individuals showed no activation of the right hemisphere mirror neuron system (MNS) during an emotion recognition and imitation task, i.e. the right pars opercularis showed significantly greater activation in typically developing children than in children with ASD. Activity in the right pars opercularis was also negatively correlated with symptom severity measured by ADOS and ADI-R. These previous reports of a right hemispheric involvement of the brain in mediating ASD symptomatology are thus in line with our findings of more significant reductions in GWC predominantly in the right hemisphere.

Little is, however, currently known about the neurobiological mechanisms that underpin variability in GWC. In general, the T1-weighted signal, which constitutes the basis of the GWC, is heavily influenced by the structure and density of axonal myelin [47, 48], as well as non-architectural components such as iron deposition and water content [49, 50]. Out of these potential candidates, studies examining cortical ageing in the TD brain show that the age-related decline in GWC is foremost related to reduced signal intensities in the superficial white matter [17] and reduced intracortical myelin content as measured by the ratio between T1w/T2w image contrast [51]. Thus, the most prominent biological candidate influencing the GWC may be the degree of myelin in the superficial WM under the cortical mantle, which mostly contains short association and U-shaped fibers [17, 42, 52]. Deficits of short association fibers have been reported previously [53] and may hence contribute to the atypical GWC observed in our study.

In addition, we examined whether the differences in GWC were driven by differences in absolute tissue intensities within the grey or white matter. In many regions with significantly reduced GWC, ASD individuals also showed significantly decreased WMI sampled at 1 mm below the grey-white matter boundary. However, there were no differences in GMI (sampled at 30% CT and at the grey-white matter boundary) compared to TD controls. Analogue to the trajectories of GWC, differences in WMI seem to be most prominent during childhood and become less pronounced during adolescence and early adulthood. This finding is in agreement with previous voxel-based-morphometry (VBM) studies in ASD that compare white matter intensity using a whole-volume approach [5, 6, 54]. Evidence for general white matter abnormalities in ASD is further supported by DTI studies applying techniques such as tract-based spatial statistics (TBSS) [55]. For example, a study by Shukla et al. [56] examined atypicalities in the trajectories of white matter development in a sample of 9- to 20-year-old ASD individuals and TD controls. Here, ASD individuals showed less orientational coherence (i.e. fractional anisotropy) and stronger water diffusion (i.e. mean diffusivity) in many of the most prominent white matter fiber tracts in the brain compared to controls [56]. These maturational differences, however, diminished from childhood to adolescence and are thus in agreement with our finding of a delayed white matter maturation (based on tissue intensities) during early childhood. Our results are thus consistent with previous publications examining cortical white matter employing different methodological frameworks and spatial scales.

Last, our findings should be interpreted in the light of a number of limitations given the data and methods presented. First, we employed a cross-sectional study design to examine age-related differences in GWC associated with ASD. Thus, the resulting age-related trajectories were based on inter- rather than intra-individual variability in GWC. Future studies are required to replicate our findings in longitudinal samples, which would provide a more accurate characterization of developmental trajectories based on repeated measures acquired in the same set of individuals. Second, our sample only included right-handed males with ASD in the high-functioning range of the spectrum. It therefore remains to be established whether our findings generalize to other (sub)groups on the autism spectrum (e.g. left-handed individuals, females with ASD, or individuals with intellectual disability). Furthermore, our study examined age-related changes in GWC by sampling tissue intensities around the grey-white matter boundary, even though this boundary may be ‘blurred’ (i.e. less well distinct) in ASD as suggested by histological evidence [12]. In this histological study, however, such microstructural blurring occured up to 500 micrometers underneath the grey-white matter transition zone [12], which would result in a maximal displacement of the boundary of 0.5 mm. As we are sampling GMI at 30% CT, which roughly equals between 0.56 and 1.5 mm into the cortical mantle depending on CT variability across the cortex (see Additional file 2), and WMI at 1 mm into the white matter, we can be certain that our sampling points remain located within the grey or white matter even in case of a maximal boundary displacement. However, while our model can accommodate such potential displacements in terms of intensity sampling, we are unable to unambiguously allocate sampling points to specific cortical layers given the restraints with regard to the current resolution of structural MRI images (see also [17]). Similarly, while surface-based mapping allows for morphometric inferences on a sub-millimeter scale, the derived grey-white matter tissue intensity values, and hence the GWC, remain dependent on the native spatial resolution of the T1-weighted images (i.e. 1 mm isotropic). Thus, partial volume effects and/or the ability to clearly delineate the grey-white matter boundary may affect GWC values. However, both of these factors are expected to affect both groups equally, and our findings of significant between-group differences and age-by-group interactions cannot be fully explained by these limitations.

## Conclusions

Using 3-Tesla structural MRI, we provide evidence that the tissue contrast at the grey-white matter boundary is regionally reduced in the brain of individuals with autism spectrum disorder. Compared to healthy controls, this neuroanatomical difference seems to be most prominent during childhood and early adolescence. Taken together, while future research is required to identify the specific neurobiological mechanisms underpinning the atypical development of tissue contrast in ASD, the findings presented in the present study suggest that the differences in GWC previously reported in adults with the condition [15] do not reflect differences in the grey matter cytoarchitecture alone. Instead, our study suggests that the differences in GWC are dynamic (i.e. ongoing) across the human life-span and are thus most likely caused by a combination of factors that include many of the grey matter atypicalities highlighted by the histological studies above [11, 12], but also by perturbations to the formation of the axonal neurocircuitry and subsequent myelination within the superficial white matter.

## Additional files

Additional file 1: Between-group differences and age-by-group interactions for GWC when including total brain volume (TBV) as a covariate. (A) Clusters with significantly reduced GWC (RFT-based, cluster corrected, *p* < 0.05) in ASD compared to controls (blue to cyan colourscale) while controlling for the effects of age and age-related interactions (i.e. main effect of group). (B) Clusters with significant linear age-by-group interactions (RFT-based, cluster corrected, *p* < 0.05). (C) Clusters with significant quadratic age-by-group interactions (RFT-based, cluster corrected, *p* < 0.05). *Note*. Significant positive age-by-group interactions are displayed in red to yellow, significant negative age-by-group interactions are displayed in blue to cyan. (PDF 1284 kb)

Additional file 2: Cumulative distribution for measures of cortical thickness (CT) across all vertices and participants. The horizontal bar shows the upper 90% of the distribution, corresponding to a CT value of 1.85 mm. (PDF 38 kb)

## Abbreviations

ADI-R: Autism Diagnostic Interview-Revised
ADOS: Autism Diagnostic Observation Schedule
ASD: Autism spectrum disorder
BA: Brodmann area
CT: Cortical thickness
DLPFC: Dorsolateral prefrontal cortex
DMN: Default mode network
DTI: Diffusion tensor imaging
FOV: Field of view
FSIQ: Full-scale intelligence quotient
FWHM: Full-width at half-maximum
GLM: General linear model
GM: Grey matter
GMI: Grey matter intensity
GWC: Grey-white matter tissue contrast
ICD: International statistical classification of diseases
IQ: Intelligence quotient
MNS: Mirror neuron system
mPFC: Medial prefrontal cortex
NU: Non-uniform
RFT: Random-field theory
TBSS: Tract-based spatial statistics
TD: Typically developing
TE: Echo time
TR: Repetition time
VBM: Voxel-based morphometry
WASI: Wechsler Abbreviated Scale of Intelligence
WM: White matter
WMI: White matter intensity
