# Supplementary material for: The effect of age on vertex-based measures of the grey-white matter tissue contrast in autism spectrum disorder
Source: Mol Autism. 2018 Oct 1;9:49. doi: 10.1186/s13229-018-0232-6 (PMC6167902; doi:10.1186/s13229-018-0232-6)
Supplement: Supplementary file 1 — Between-group differences and age-by-group interactions for GWC when including total brain volume (TBV) as a covariate. (A) Clusters with significantly reduced GWC (RFT-based, cluster corrected, p < 0.05) in ASD compared to controls (blue to cyan colourscale) while controlling for the effects of age and age-related interactions (i.e. main effect of group). (B) Clusters with significant linear age-by-group interactions (RFT-based, cluster corrected, p < 0.05). (C) Clusters with significant quadratic age-by-group interactions (RFT-based, cluster corrected, p < 0.05). Note. Significant positive age-by-group interactions are displayed in red to yellow, significant negative age-by-group interactions are displayed in blue to cyan. (PDF 1284 kb) [file 13229_2018_232_MOESM1_ESM.pdf]

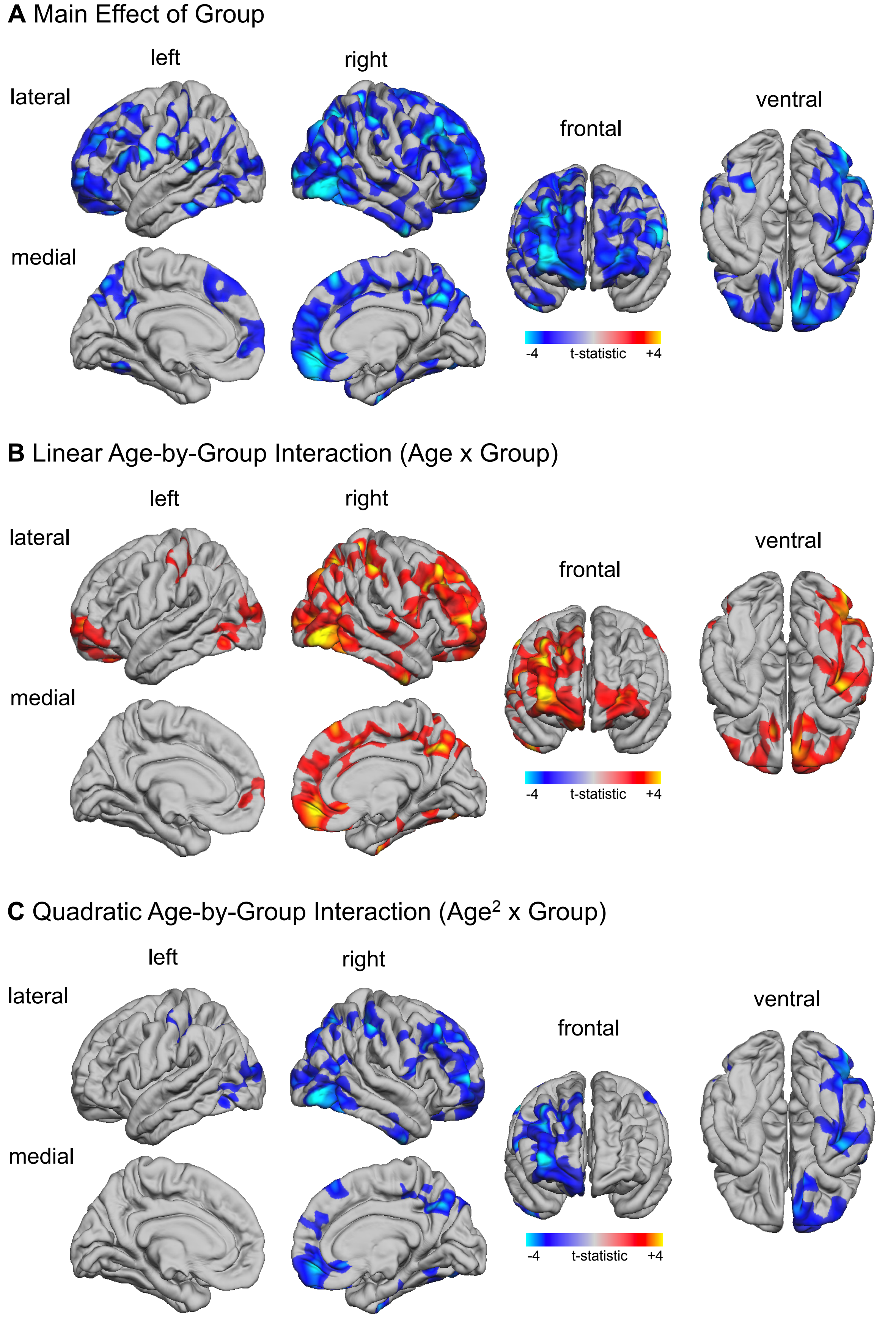
A Main Effect of Group
left
right
lateral
medial
frontal
ventral
-4
t-statistic
+4
B Linear Age-by-Group Interaction (Age x Group)
left
right
lateral
medial
frontal
ventral
-4
t-statistic
+4
C Quadratic Age-by-Group Interaction (Age2 x Group)
left
right
lateral
medial
frontal
ventral
-4
t-statistic
+4
