# Supplementary figures and images for: The effect of age on vertex-based measures of the grey-white matter tissue contrast in autism spectrum disorder
Source: Mol Autism. 2018 Oct 1;9:49. doi: 10.1186/s13229-018-0232-6 (PMC6167902; doi:10.1186/s13229-018-0232-6)

Empirical CDF

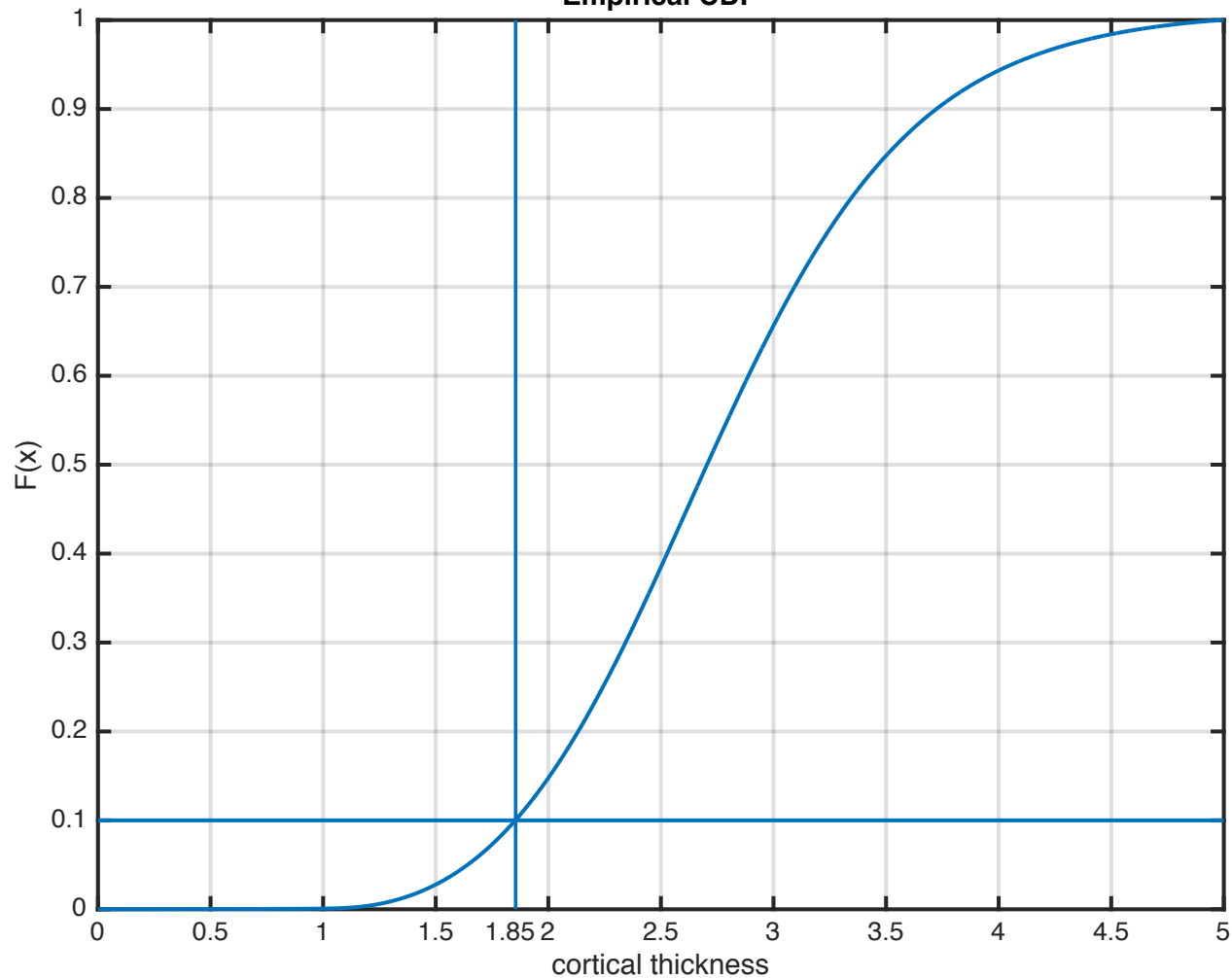

Supplement: Supplementary file 2 — Cumulative distribution for measures of cortical thickness (CT) across all vertices and participants. The horizontal bar shows the upper 90% of the distribution, corresponding to a CT value of 1.85 mm. (PDF 38 kb) [file 13229_2018_232_MOESM2_ESM.pdf]
